# Rituximab in refractory sarcoidosis: a single centre experience

**DOI:** 10.1186/s12948-015-0025-9

**Published:** 2015-09-01

**Authors:** Francesco Cinetto, Nicolò Compagno, Riccardo Scarpa, Giacomo Malipiero, Carlo Agostini

**Affiliations:** Clinical Immunology, Department of Medicine-DIMED, Padova University Hospital, via Giustiniani 2, 35128 Padua, Italy

**Keywords:** Refractory sarcoidosis, Rituximab, Granulomatosis

## Abstract

Sarcoidosis is a granulomatous disease whose outcome varies from spontaneous remission to chronic refractory disease. Provided that steroids represent the gold standard as a first line treatment, many immune suppressants drugs are currently used in the disease management. However, refractory disease is still a great challenge. Rituximab is an anti-CD20 chimeric monoclonal antibody, currently used for the treatment of B cell malignancies and systemic autoimmune diseases. There are few case reports describing the successful use of Rituximab in refractory sarcoidosis with lung, eye, lymph nodes and skin involvement. In this paper we described three different case reports in which Rituximab has been used to treat refractory sarcoidosis and we reviewed the existing literature.

## Background

Sarcoidosis is a granulomatous disease whose outcome is quite variable. Most patients undergo spontaneous resolution or present a good response to systemic treatment, but a small percentage of patients develop a chronic, progressive disease, which can be refractory to multiple lines of treatment [1]. Provided that steroids represent the gold standard as first line treatment, immunosuppressive drugs and antimalarial therapies are currently used in the clinical management of the disease, when steroid-dependence or resistance develop. Over the last few years, anti-TNFα agents have become a therapeutic option in refractory sarcoidosis [2]. However, there is still need for further options for those patients whose disease fail to respond to the above mentioned drugs.

Despite being a T cell mediated disease [3], humoral mechanisms might play a role in the pathogenesis of sarcoidosis [4]; moreover, B-cell-targeted therapies have been successfully used in autoimmune disorders in which T cells has been demonstrated to have a prominent pathogenetic role [5, 6].

Rituximab is an anti-CD20 chimeric monoclonal antibody, currently used for the treatment of B cell malignancies and immune-mediated diseases such as rheumatoid arthritis and ANCA-associated vasculitides [7–10]. Few case reports and a retrospective study suggested the effectiveness of rituximab in the management of refractory sarcoidosis [11–15]. A prospective phase I/II trial has also been published investigating the role of rituximab in the treatment of refractory pulmonary sarcoidosis [16].

Herein we describe the impact of Rituximab in three diverse cases of refractory sarcoidosis with different organs involvement and we review the currently available evidence regarding the use of the anti-CD20 monoclonal antibody as a therapeutic option in this granulomatous disease.

## Cases presentation

### Case 1

A 35 years old Caucasian male presenting painful bilateral enlargement of inguinal lymph nodes and progressive dyspnoea underwent node biopsy, allowing a histologic diagnosis of sarcoidosis. A chest X-ray was consistent with a stage 2 sarcoidosis according to Scadding classification (mediastinal lymphadenopathy and interstitial pulmonary involvement), confirmed by a subsequent CT scan, also revealing abdominal lymphadenopathy. Pulmonary function tests showed a mild restrictive disease and a moderate reduction in DLco value; bronchoalveolar lavage fluid analysis demonstrated increased number of lymphocytes and a high CD4/CD8 T-lymphocytes ratio. Ophthalmologic evaluation was unremarkable and no skin lesions were detected.

Due to symptomatic pulmonary involvement, patient was initially treated with corticosteroids, starting with prednisone 30 mg/day (0.5 mg/kg of body weight) in June 2008, progressively tapered until 5 mg/day in September 2009. CT scan and pulmonary function tests both displayed an improvement after therapy, with normalization of lymph nodes size, recovery of interstitial pulmonary disease and increase of FVC with remission of dyspnoea; DLco was stable. Unfortunately, patient remained steroid-dependent and, early in 2010, developed a recrudescence of dyspnoea. Therapy with methotrexate (10 mg/week) was therefore started, obtaining a new remission of symptoms. 6 months later patient was asymptomatic; CT scan only revealed mild fibrotic scars interesting the upper lobes and pulmonary function tests were normal, with exception of a slight DLco decline. Thus, methotrexate was progressively tapered and stopped in 6 months time.

**Fig. 1:**
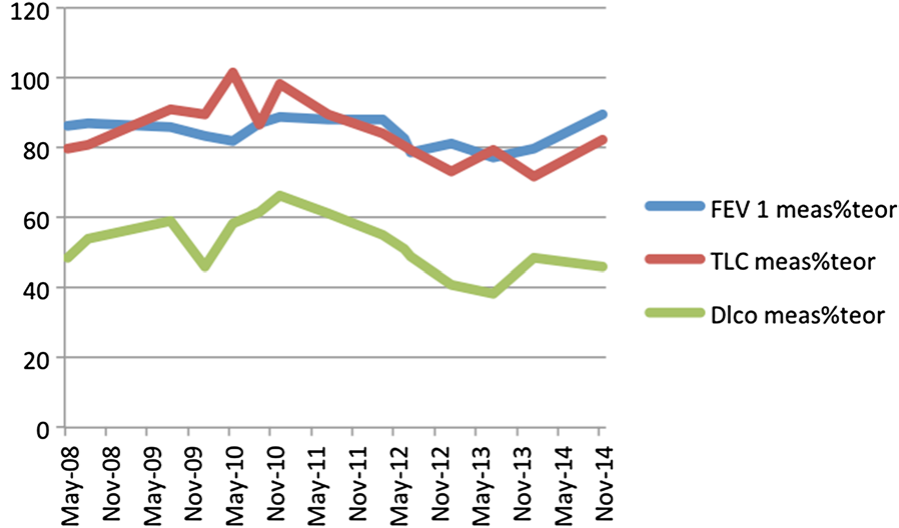
Lung volumes and DLco in patient 1.

Eight months later patient complained of a new worsening of dyspnoea and a CT scan showed an interstitial pulmonary involvement, prevalent in the upper lobes, with recurrence of lymphadenopathy. Azathioprine was started and suspended in few weeks, due to intolerance. Cyclophosphamide was then administered every 2 weeks in our outpatient clinic for 6 times; the initial dose was 600 mg, later escalated up to 750 mg. A chest CT scan performed at the end of the treatment showed no progression of the disease, but a new assessment after 6 months revealed new radiological signs of active interstitial lung disease, extending from upper to lower lobes, with increasing evidence of parenchymal and peri-bronchial fibrosis. A worsening was registered in terms of dyspnoea and lung function (Fig. 1). Patient refused infliximab as a treatment option, due to the long term administration schedule. Thus, rituximab 375 mg/m^2^ every 2 weeks for six times was administered. Treatment was well tolerated and three further evaluations, respectively 6, 12 and 18 months after the end of the treatment, highlighted an arrest in lung disease progression. At present patient only has radiological evidence of inactive fibrotic lesions, prevalently located at upper lobes, and a stable restrictive disease (Fig. 1); he is under maintenance treatment with hydroxychloroquine.

### Case 2

Patient 2 was referred to our outpatient clinic in June 2013, due to chronic sarcoidosis refractory to standard therapy. He was a North-African 44 years old man, employed in a tannery. The past medical history included papillary breast cancer, hypertension (treated with calcium channel blockers) and paroxysmal atrial fibrillation. The breast cancer was diagnosed in January 2008 and treated with radical mastectomy and subsequent hormone therapy with tamoxifene up to March 2013, when last oncologic evaluation showed no evidence of relapse after 5 years. Serologic tests indicated a previous HBV infection.

The first report of granulomatous disease was dated at the time of mastectomy, when histological examination of sentinel lymph nodes resulted negative for cancer infiltration, but described the presence of a granulomatous reaction. A chest X-ray then showed bilateral mediastinal enlargement, with no metabolic activity at a ^18^F-FDG PET/CT scan. At that time patient was asymptomatic and a follow-up plan was started as for an asymptomatic stage 2 sarcoidosis.

One year later, in 2009, inguinal lymph node enlargement became clinically evident, and a PET/CT scan showed marked FDG uptake in mediastinal, carenal, axillary and inguinal lymph nodes. A cutaneous lesion appeared involving the nose, classified as *lupus pernio*. Nodal and cutaneous biopsies showed non-necrotizing granulomatous inflammation, consistent with sarcoidosis. Patient did not present systemic symptoms at that time point. Specific therapy for sarcoidosis was then started, but clinical signs of the disease did not improve after corticosteroid treatment (prednisone) not even when associated to azathioprine. The patient, conversely, started complaining of fever and cough and azathioprine was replaced by methotrexate and high dose steroids (1 mg/kg, then tapered), with initial benefit but recurrence of systemic symptoms when tapering the steroids below 10 mg/die. A PET-CT scan (April 2013) was superimposable to the previous one, in terms of lymph nodes involvement, but showing an increased metabolic activity.

Due to the refractoriness of his disease, with a high-dose steroid dependence and persistence of systemic symptoms (fever, fatigue, cough, dyspnoea on exertion) the patient was at this point referred to our outpatient clinic. Physical examination was normal, except for the presence of *lupus pernio* and one cutaneous lesion on the forehead. ACE levels were normal. Pulmonary function tests showed a slight reduction of lung volumes with normal flow rates; diffusion of CO was reduced (67%).

Given the recent anamnestic malignancy and after discussion with the Oncologists, we decided to avoid anti-TNF drugs and to start a treatment with Rituximab at the dose of 1 g per month (this schedule was necessary to match patient’s travel needs). Hydroxychloroquine was started as a co-treatment, progressively tapering the prednisone. After 3 monthly infusions, symptoms improved despite the complete steroids withdrawal: fever disappeared, cutaneous lesions were significantly reduced compared to baseline, and only a slight dyspnea during exercise persisted. Pulmonary function tests and a PET-CT scan were superimposable to the tests performed before Rituximab; ACE levels were abnormally increased. 4 months after the last dose systemic symptoms recurred, associated with exacerbation of the cutaneous lesions. Three monthly administrations of Rituximab were repeated, followed by a maintenance treatment with mycophenolate mofetil. During this second course of treatment, we observed only a slight and temporary improvement in cutaneous lesions.

At present, after 8 months of therapy with mycophenolate mofetil, the clinical picture is worsening: the patient is still complaining of fatigue and dyspnoea on exertion, cutaneous lesions are slowly worsening; PET/CT scan picture is superimposable to that performed before the first Rituximab course. Diffusion of CO is further reduced (48%), while lung volumes and flow rates are stable. We are currently evaluating other therapeutic options.

### Case 3

A 29 years old Caucasian woman was referred to our ILD outpatient clinic in April 2001; she had a 6 years history of pulmonary, cutaneous and ocular sarcoidosis, previously treated with two different lines of therapy: deflazacort/methotrexate and deflazacort/azathioprine. Physical examination showed two violaceous-erythematous, non-itchy, non-painful nodules over the knees. A *fundus oculi* examination detected residual atrophic granulomatous lesions, bilaterally. No hilar enlargement or interstitial lung disease were detected by a chest X-ray. High resolution computed tomography showed mild signs of diffuse interstitial lung disease, with preserved respiratory volumes and DL_CO_ at pulmonary function tests. Routine and immunological blood test results were normal. We confirmed the ongoing therapy with deflazacort and azathioprine, started 10 months before. From March 2001 to August 2006 the patient was lost to follow-up; during these years she underwent a progressive worsening of visual acuity. In August 2006, following the spontaneous attempt to taper her steroid therapy, a recurrence of severe cutaneous and ocular signs of disease brought the patient back to medical attention. Erythematous lesions were present on her knees at clinical examination; an ophthalmologic investigation showed bilateral retinal granulomatous disease and vasculitis, with more serious involvement of the right eye; chest HRCT and pulmonary function tests showed no worsening of lung disease; a complete blood tests panel was unremarkable. A 67-Ga scintigraphy underlined mild hypermetabolism in mediastinal nodes and lacrimal glands, bilaterally. A new treatment schedule including infliximab (5 mg/kg every 6 weeks for a total of 6 infusions), methotrexate (15 mg/week) and deflazacort (3 mg/die) achieved an almost complete clinical response; thus, a maintenance therapy was established with infliximab 5 mg/kg every 8 weeks, methotrexate 7,5 mg/week and deflazacort 1.5 mg/die. Steroids were completely withdrawn in September 2011. A chest CT in June 2011 showed marked reduction of the mediastinal lymphadenopathy.

In February 2012, during a follow-up visit, a further recurrence of ocular and cutaneous disease was detected. Thus, patient was shifted to adalimumab (40 mg every 2 weeks) and prednisone (10 mg/die), with only partial response. In October 2012 disease remission was achieved using the following schedule: cyclophosphamide (6 administrations of 500 mg, once weekly), mycophenolate mofetil (MM, 1000 mg/day) and prednisone (12.5 mg/day). Unfortunately, eye and skin disease recurred early during the maintenance treatment with mycophenolate mofetil and prednisone. At that point, weekly rituximab treatment (6 administration of 375 mg/m^2^, once weekly) was established. Since ocular disease was still progressing after the third infusion, cyclophosphamide was added to treatment (700 mg/administration for 5 administrations every 2 weeks). Unfortunately, disease progression was not arrested and patient complained of complete sight loss of the right eye. Left eye examination showed vitreitis and active retinal granulomatosis and vasculitis. Given the high risk of complete sight loss and hypothesizing an activity of the anti-CD20 treatment on those B cells sustaining the possible anti-Infliximab antibody production, we made a new attempt of treatment with Infliximab. This attempt was surprisingly successful, achieving in few weeks a complete resolution of skin lesions and a satisfactory control of the ocular disease. Patient has currently no active disease under maintenance treatment with infliximab and a minimum dose of prednisone.

## Conclusions

This small case series analyses the impact of Rituximab on three different settings of chronic and refractory sarcoidosis. Moreover, the administration schedule and dosage were not homogeneous, as well as the degree of effectiveness.

Patient 1 presented a chronic pulmonary sarcoidosis, with significant impairment of lung function; Rituximab was administered at a dosage of 375 mg/m^2^ every 2 weeks for six times, arresting the disease progression. 18 months after treatment clinical and radiological pictures are still stable and this is the longest period of disease quiescence since diagnosis. Patient number 2 had a skin, lung and nodal disease, and the concomitant malignancy strongly influenced the treatment approach for sarcoidosis. Administration schedule has been in turn influenced by patient’s needs for travelling, the dose was 1 g per infusion monthly for three consecutive months; treatment was at first effective, but had to be repeated in 6 months due to early recurrence of symptoms and the second course was not as effective. The main goal achieved, in this case, was the withdrawal of steroids after years of treatment. The third patient presented an ocular disease with rapidly progressive sight loss; thus, a more dose-intensive lymphoma-like schedule was adopted (375 mg/m^2^/administration for six consecutive weeks) and cyclophosphamide was associated after 3 of 6 anti-CD20 MAb infusions. In this patient rituximab was not effective on sarcoidosis activity, but we may hypothesize that anti-CD20 treatment was able to disrupt a possibly B-cell dependent resistance to infliximab, thus somehow contributing to the disease management.

In terms of safety, no one of the administration schedules induced any infusion-related adverse events. Infusion pre-treatment included paracetamol, antihistamines and dexamethasone, as standard practice, and patient 2 received a Lamivudine prophylaxis during the months of treatment and for 6 further months, due to serologic markers of anamnestic HBV infection. All patients presented a peripheral blood CD19 cells depletion 3 months after the last anti-CD20 infusion. No lymphopenia was detected before or after treatment. No relative or absolute hypogammaglobulinemia occurred considering a mean of 1.2 years of follow-up after treatment. In particular, all patients showed mild serum hypergammaglobulinemia before the first rituximab administration, that was no more detectable 3 months later. Gamma globulin levels never decreased below the reference value during follow-up; IgG, IgA and IgM levels were not significantly affected at any time point. Onset of therapeutic effects, if any, occurred within one month after the first administration.

In terms of efficacy, it is not possible to draw any definitive conclusion on the basis of these three case reports that, indeed, represent a good sample of the previously published reports on this item. To our knowledge, there are only case reports, one retrospective study and a phase I/II open label trial currently available in literature, discussing the effectiveness of Rituximab in refractory sarcoidosis (Table 1). Single case reports discussed successful use of anti-CD20 treatment in one case of neurosarcoidosis [11], one of sarcoidosis-associated neuromyelitis optica [12], one of lung, lymph-nodes and joint disease [15], one with peritoneal nodes involvement [13] and one more case of lymph node disease included in a case series of systemic autoimmune diseases [17]. The retrospective study included 4 patients with refractory ocular sarcoidosis, describing good results for three of them [14]. Finally, the prospective study, involving ten patients with chronic lung disease with moderate to severe pulmonary disease still symptomatic despite using a combination of corticosteroids and steroid sparing agents, showed inconsistent results within this small cohort of patients [16]. Thus, if single case reports point at rituximab as an effective treatment option in refractory disease, when moving to case series the matter appears to be more controversial. Our case series is in line with this consideration, inasmuch we observed clear benefit only in one of three patients. This also suggests the need for identifying parameters that could be suggestive for a putative good response in sarcoid patients with refractory disease.

**Table 1:** Summary of reported case records and retrospective/prospective studies

References	Main organ involvement	Dose	Outcome
Gottenberg et al. [17]	Lymph nodes	4 × 375 mg/m^2^	Good
Belkhou et al. [15]	Lymph nodes	2 × 1 g, every 2 weeks	Good
Dasilva et al. [13]	Lymph nodes, including peritoneal	Two infusions, dose not specified	Good
Bomprezzi et al. [11]	CNS and skin	2 × 1 g, every 2 weeks, then 1 g every 6 months for 2 years	Good
Lower et al. [14]	Eye; 2 of 4 patients also had significant lung involvement	2 × 1 g, every 2 weeks, then 375 mg/m^2^ every 4 or 8 weeks for a minimum of 24 months or 1 g every 4 weeks for 10 months	Three responders of four patients
Sawaya et al. [12]	Neuromyelitis optica and lymph nodes	Single 1 g infusion	Good
Sweiss et al. [16]	Lung	2 × 1 g, every 2 weeks	Seven full or partial responders of ten patients

Moreover, it is noteworthy that there is no agreement on the administration schedule or dosage, with a split between the lymphoma-like approach (375 mg/m^2^/administration every one or two weeks for 4–6 total administration) and a more Rheumatoid Arthritis-oriented schedule (1 g every 2 weeks, for 2 or more administrations). Almost all patients have also been co-treated with different immune-suppressants and a long-term treatment with other drugs has been established after rituximab. The ophthalmologic case series also provides data on an anti-CD20 maintenance treatment, which is currently the standard of care in many hematologic malignancies, with a minimum duration of 10 months but different schedules. In addition, as expected in refractory diseases, the treatment history before rituximab of all the reported patients is extremely heterogeneous and it would be really hard to define which patients to include in a perspective study, also considering that last ATS/ERS/WASOG guidelines on sarcoidosis are dated 2009.

Nevertheless, in consideration of some good results described in cases with poor prognosis, particularly in eye disease, all these open questions (patient selection, dose and administration schedule, co-treatment and maintenance treatment) would deserve an evidence based answer. Thus, there is a strong need for larger case series and prospective studies, in order to possibly design a multicentre prospective trial, the only way to get definitive answers and to optimize the use of health-care resources.

Finally, the rationale and mechanism of action of an anti-CD20 treatment in this T-cell mediated granulomatous disease remains an intriguing topic, strongly related to what we still do not understand of etiology and pathogenesis of this granulomatous disease.

## Consent

Written informed consent was obtained from the patient for publication of this Case Report and any accompanying images. Patient 2, due to personal and religious concerns, did not allow us to take any picture of his skin lesions. A copy of the written consent is available for review by the Editor-in-Chief of this journal.
